# Editorial: Synthesis and Bioactivities of Plant-Derived Biomolecules

**DOI:** 10.3389/fpls.2022.949057

**Published:** 2022-06-24

**Authors:** Guiguo Zhang, Yunkyoung Lee, Zeng-Yu Wang, Yuxi Wang

**Affiliations:** ^1^Department of Animal Nutrition, China-Korea Joint R&D Center on Plant-Derived Functional Polysaccharide, Shandong Agricultural University, Taian, China; ^2^Department of Food Science and Nutrition, Korea-China Joint R&D Center on Plant-Derived Functional Polysaccharide, Interdisciplinary Graduate Program in Advanced Convergence Technology and Science, Jeju National University, Jeju, South Korea; ^3^College of Grassland Science, Qingdao Agricultural University, Qingdao, China; ^4^Lethbridge Research and Development Centre of Agriculture and Agri-Food Canada, Ottawa, ON, Canada

**Keywords:** plant, compound, biomolecule, bioactivities, biosynthesis

There are various bioactive compounds in plants, including polysaccharides, polyphenols, anthocyanins, terpenoids, carotenoids, and other basic chemical compounds and/or secondary metabolites, which play important roles in plant growth, development, and resistance to stresses (Zaynab et al., 2018). The biosynthesis, metabolism, and accumulation of these compounds in plants are intimately associated with species, varieties, growth stages, organ, tissues, ecological environment, and biotic or abiotic stresses (Zhao et al., 2021; Kim et al., 2022). A full understanding of the metabolic pathways, structural characteristics, and biological functions of these active substances in plants are of great significance for elucidating plant stress-resistant physiology, crop yield and quality trait formation (Ghassemi et al., 2021). In addition, when those compounds are consumed by human and/or animals, they exhibit multiple bioactivities to affect host physiological metabolism and health mainly through interaction with cells and/or gut microbes. Extensive research has been conducted on the biosynthesis regulation of these plant compounds and their roles in plant physiology (Mitra et al., 2021). Recently, there is increasing interest in the nutraceutical and/or pharmaceutical usage of these compounds in human and animal due to the increasing restrictions on the use of antibiotics in humans and global bans on the prophylactic and growth-promoting use of antibiotics in animal food production (Pan et al., 2022).

A total of seven innovative articles have been published in this Research Topic of “*Synthesis and Bioactivities of Plant-Derived Biomolecules*” which represent the latest findings in this multi-disciplinary research area. Four of these publications focused on factors associated with the biosynthesis and metabolism of specific bioactive compounds in plants, and the other three elucidated the chemical composition and structural characteristics of some specific plant bioactive compounds and defined their novel bioactivities.

Glucosinolates (GSLs) are secondary metabolites in cruciferous plants, which play an important defensive role against the attacks of pathogenic fungi and insects (Kim et al., 2022). In addition, GSLs determine the flavor, taste, and quality trait of the plants and possess anti-cancer properties. Feng et al. analyzed the varied contents of GSLs in leaf, stalk, and flower bud of Chinese flowering cabbage and documented that the biosynthesis, transportation, and accumulation of GSLs in these tissues were positively correlated to the related gene expressions. In another parallel study, Frerigmann et al. discovered that the key repressor of the light signaling, the constitutive photomorphogenic1/suppressor of phytochrome a-105 (cop1/spa) complex, was a crucial component to regulation of GSL biosynthesis. Similarly, Chen et al. revealed that the biosynthesis of terpenoids in the roots of *Rehmannia glutinosa* were controlled by multiple genes, and was significantly impacted by the varieties, phenological stages, or different parts of the plant. Huang et al. also reported that concentration and chemical constituents of condensed tannins (CT) in purple prairie clover (PPC, *Dalea purpurea* Vent.) varied in different growth stages and tissues, possibly influenced by changes in gene expressions during plant growth and development. Therefore, understanding of related genes controlling compound biosynthesis and metabolism in plants is pivotal for developing new methods in molecular breeding and for creating new germplasm resources. Zhou et al. observed that some flavonoid metabolites involved in quercetin, tricin, and rutin metabolism in *Agriophyllum squarrosum (L.) Moq*. were significantly enriched in the low-altitude populations than those populations from middle and high altitude, indicating that environmental stress may affect accumulation of flavonoid metabolites more than genetic differentiation in *A. squarrosum*. This information gave us an insightful perspective that the biosynthesis and metabolism of some plant compounds could be modulated by manipulating the ecological environment of the plant.

Polysaccharides are plant primary metabolites and are commonly deposited in the cytoplasm as energy storage materials or in the cell walls as structural components (Cui et al., 2022). The leaf cell walls of chicory (*Cichorium intybus*) are known to contain high proportions of pectic polysaccharides. Sun et al. investigated the distribution of pectic polysaccharides in the cell walls of chicory leaves using immunolabelling techniques with four monoclonal antibodies and found differential distribution of pectic epitopes among walls of different cell types and within the cell walls, which may reflect that the deposition and modification of these polysaccharides are involved in cell wall properties and cell development.

Some biomolecules synthesized in plants may exert multiple beneficial or detrimental bioactivities to humans/animals (Ahmed et al., 2022). The α-chaconine is the most abundant glycoalkaloid in potatoes and is toxic to the human/animal digestive system. He et al. observed that α-chaconine disrupted the cell cycle, destroyed the mechanical barrier and permeability of intestinal mucosal epithelium, inhibited cell proliferation, and accelerated cell apoptosis (*in vitro*). Similarly, CT are secondary plant metabolites that are widely present in in plants. They possess varying biological, pharmaceutical and nutraceutical activities such as antimicrobial, antioxidant, anti-cancer, anti-inflammatory, anti-diabetic, and immune-promoting effects, these activities are closely related to their origin (chemical composition) and dietary concentration of CT. Huang et al. for the first time elucidated the chemical and structural composition of CT in PPC in different growth stages and from different plant parts. The results demonstrated that the concentration and chemical composition of CT varied in different tissues and different maturities, which in turn affected their biological activities. The mean degree of polymerization was the highest for CT in stems and increased with maturity, while CT isolated from leaves at the early flowering head stage exhibited the greatest protein precipitation capacity.

In conclusion, the biosynthesis of biomolecules in the plant was regulated by specific genes as well as the external ecological environment. These bioactive compounds play a vital role in enhancing resistance to abiotic/biotic stress, and the formation of quality traits in plants. An in-depth understanding of those crucial factors that define biosynthesis and structural characteristics of these biomolecules in the plant would facilitate the employment of molecular breeding techniques to specifically develop plant varieties rich in certain biomolecules and with enhanced beneficial bioactivities.

## Author Contributions

The idea of and concept of this Research Topic came out from the discussion among the guest editors. GZ and YL finalized the writing of this Editorial. YW and Z-YW gave some valuable suggestions. All authors contributed to the writing process of the Editorial and approved the final version of it.

## Funding

This work was supported by the National Key R&D Program of China-Korea Cooperative Project (2019YFE0107700 and NRF-2019K1A3A1A20081146), the National Research Foundation Grant of Korea (NRF-2017R1D1A3B03031665 and NRF-2020R1A2C2004144), the Forage Industrial Innovation Team Project (SDAIT-23-05), Shandong Province Technology Innovation Guidance Plan (2019YFE0107700), and the Excellent Seed Project of China (2019LZGC012).

## Conflict of Interest

The authors declare that the research was conducted in the absence of any commercial or financial relationships that could be construed as a potential conflict of interest.

## Publisher's Note

All claims expressed in this article are solely those of the authors and do not necessarily represent those of their affiliated organizations, or those of the publisher, the editors and the reviewers. Any product that may be evaluated in this article, or claim that may be made by its manufacturer, is not guaranteed or endorsed by the publisher.
